# Dietary Consumption of Phenolic Acids and Prostate Cancer: A Case-Control Study in Sicily, Southern Italy

**DOI:** 10.3390/molecules22122159

**Published:** 2017-12-05

**Authors:** Giorgio Ivan Russo, Daniele Campisi, Marina Di Mauro, Federica Regis, Giulio Reale, Marina Marranzano, Rosalia Ragusa, Tatiana Solinas, Massimo Madonia, Sebastiano Cimino, Giuseppe Morgia

**Affiliations:** 1 Urology Section, University of Catania, 95124 Catania, Italy; daniele-campisi1987@virgilio.it (D.C.); marinadimauro@live.it (M.D.M.); federicaregis23@gmail.com (F.R.); dottorgreale@gmail.com (G.R.); ciminonello@hotmail.com (S.C.); gmorgia@policlinico.unict.it (G.M.); 2 Department of Medical and Surgical Sciences and Advanced Technologies “G.F. Ingrassia”, Section of Hygiene and Preventive Medicine, University of Catania, 95124 Catania, Italy; marranz@unict.it; 3 Health Direction of Policlinic Hospital, 95100 Catania, Italy; ragusar@unict.it; 4 Urology Section, University of Sassari, 07100 Sassari, Italy; tatiana.solinas@libero.it (T.S.); massimomadonia@gmail.com (M.M.)

**Keywords:** diet, Mediterranean diet, phenolic acids, prostate cancer, risk factors

## Abstract

Dietary polyphenols gained the interest of the scientific community due to their wide content in a variety of plant-derived foods and beverages commonly consumed, such as fruits, vegetables, coffee, tea, and cocoa. We aimed to investigate whether there was an association between dietary phenolic acid consumption and prostate cancer (PCa) in South Italy. We conducted a population-based case-control study from January 2015 to December 2016 in a single institution of the municipality of Catania, southern Italy (Registration number: 41/2015). Patients with elevated PSA and/or suspicious PCa underwent transperineal prostate biopsy. A total of 118 histopathological-verified PCa cases were collected and a total of 222 controls were selected from a sample of 2044 individuals. Dietary data were collected by using two food frequency questionnaires and data on the phenolic acids content in foods was obtained from the Phenol-Explorer database (www.phenol-explorer.eu). Association between dietary intake of phenolic acids and PCa was calculated through logistic regression analysis. We found lower levels of caffeic acid (2.28 mg/day vs. 2.76 mg/day; *p* < 0.05) and ferulic acid (2.80 mg/day vs. 4.04 mg/day; *p* < 0.01) in PCa when compared to controls. The multivariate logistic regression showed that both caffeic acid (OR = 0.32; *p* < 0.05) and ferulic acid (OR = 0.30; *p* < 0.05) were associated with reduced risk of PCa. Higher intake of hydroxybenzoic acids and caffeic acids were associated with lower risk of advanced PCa. High intake of caffeic acid and ferulic acid may be associated with reduced risk of PCa.

## 1. Introduction

Prostate cancer (PCa) represented the most common incident cancer in men in developed countries in 2013 [1]. The number of incident cases increased more for PCa than for any other malignancy, irrespective of development status. Incidence and death rates have risen considerably between 1990 and 2013, with the steepest rise in age-standardized incidence rates of all cancers in men, and a higher percent change in developing versus developed countries [1]. Interestingly, epidemiological studies have shown strong evidence for a genetic predisposition to PCa, based on two of the most important factors, racial/ethnic background and family history [2]. However, findings indicated that some exogenous factors may influence the risk of progression from latent to clinical PCa. Factors such as diet, sexual behavior, alcohol consumption and others have been underlined thanks to their potential implication in the pathogenesis of various cancers [3,4].

Among different compounds, dietary polyphenols gained a particular interest due to their wide content in a variety of plant-derived foods and beverages commonly consumed, such as fruits, vegetables, coffee, tea, and cocoa [5]. These compounds are the widest family of phenolic molecules existing in nature, which are grouped according their chemical structures in flavonoids and non-flavonoids, such as phenolic acids, stilbenes, lignans, alkyphenols, and others [6]. Phenolic acids are divided into the two major classes hydroxybenzoic and hydroxycinnamic acids. Each class may contain up to hundreds of individual compounds that have been demonstrated, to a various extent, to regulate several cellular molecular pathways leading to anti-oxidative, anti-proliferative, and anti-inflammatory effects [6]. Polyphenol-rich foods, such as coffee and tea, have demonstrated substantial association with decreased risk of cancer mortality [7]. In particular, growing evidence suggested that coffee may play an important role in cancer prevention [8]. Research on polyphenols has widely grown over the last years, providing the rationale for the potential beneficial effects of these compounds and polyphenol-rich foods [9]. However, the vast majority of research has been conducted on flavonoids, while epidemiological data on phenolic acids (highly contained in coffee) are scarce. Thus, the aim of this study was to investigate whether there was an association between dietary phenolic acid consumption and prostate cancer in a sample of southern Italian individuals. 

## 2. Results

Table 1 lists the baseline characteristics of the patients. In the total cohort of 340 patients, 118 were cases of PCa (34.7%) and 222 were controls (65.3%). There were no significant differences between cases and controls, with the exception for family history of cancer and low educational status, which were more frequent among cases.

Mean consumption of phenolic acids and main subclasses and individual compounds are shown in Table 2. We found lower levels of caffeic acid (2.28 mg/day vs. 2.76 mg/day; *p* < 0.05) and ferulic acid (2.80 mg/day vs. 4.04 mg/day; *p* < 0.01) in PCa when compared to controls. 

The energy-adjusted logistic regression analysis showed that high intake of caffeic acid (OR = 0.28 (95% CI: 0.13–0.58)) and ferulic acid (OR = 0.50 (95% CI: 0.26–0.97)) were associated with reduced risk of PCa (Table 3). 

The multivariate logistic regression adjusted for age, energy intake, weight status, smoking status, alcohol consumption, physical activity level, family history of prostatic cancer, confirmed previous results, being both caffeic acid (OR = 0.32 (95% CI: 0.11–0.87)) and ferulic acid (OR = 0.30 (95% CI: 0.10–0.85)) associated with reduced risk of PCa (Table 3). No other significant association between individual phenolic acids and PCa risk was found. When analysis was restricted to advanced PCa only, higher intake of hydroxybenzoic acids and caffeic acids were significantly associated with lower risk (Table 4).

## 3. Discussion

In this study, we investigated the association between phenolic acids intake and PCa incidence. We found that high intake of caffeic acid and ferulic acid were significantly associated with reduced risk of PCa. Based on our knowledge, the present study is the first case-control investigating the relation between dietary phenolic acid intake and PCa in a Mediterranean region.

In the last decades, there has been an increased interest in preventing PCa thanks to its low progression and long latency. Previous data about diet or nutrient prevention of PCa showed contrasting results between diet and prevention of PCa [10,11,12,13]. However, great interest has been paid to dietary polyphenols, which have been investigated for their potential antitumor effects due their anti-oxidant and anti-inflammatory characteristics. Most research has been focused on phystoestrogen and flavonoids, which showed promising results in potentially preventing PCa [14]. Among others, phenolic acids are of great interest because foods rich in these molecules, such as coffee and tea, have demonstrated an association with decreased risk of PCa [15,16]. However, tea is not commonly consumed in Southern Europe, while major sources of phenolic acids have been reported to be nuts and other foods linked to the Mediterranean dietary pattern [17]. There is evidence of association between nut consumption and decreased risk of cancer and mortality, but not specific relevant finding specific on PCa [18]. In both cases, the main mechanisms of action related to the potential protective effects of such foods involve their content in antioxidants and their anti-inflammatory properties, mostly entirely related to their polyphenolic component [19,20,21]. Interestingly, no previous observational studies have been conducted to test potential associations between phenolic acids consumption and PCa. In our study, we found that only individual phenolic acids, such as ferulic acid and caffeic acid, were inversely associated with PCa. Ferulic acid (4-hydroxy-3-methoxycinnamic acid) is a phenolic compound abundant in fruits, vegetables, cereals and coffee [22]. Ferulic acid has been demonstrated to inhibit cell proliferation and to decrease oncogene expression in cell line of lymph node carcinoma of the prostate (LNCaP) and PC-3 cells [22]. Caffeic acid is a nuclear factor 'kappa-light-chain-enhancer of activated B-cells (NF-κB) inhibitor at concentrations of 50 μM to 80 μM by preventing the translocation of p65 unit of NF-κB and the binding between NF-κB and DNA [23]. Caffeic acid treatment decreased Skp2 and Akt1 protein expression in LNCaP tumors as compared to control group [24]. NF-kB/relA transcription factor is constitutively activated in human PCa cells and inhibition of NF-κB activity in PCa cells associates with suppression of angiogenesis, invasion, and metastasis. IκBα (inhibitor of kappa B) inactivates the transcription of NF-κB by masking the nuclear localization signals of NF-κB proteins and thus keeps them inactive in the cytoplasm. All these mechanisms could be the links between caffeic acid and ferulic acid intake and PCa incidence reduction. There are also some indirect mechanisms related to these compounds and their major food-sources. For instance, coffee and nuts consumption has been related with better metabolic status, lower BMI and less occurrence of cardio-metabolic risk factors [25,26], which in turn may be mediating effects for the decreased risk of cancer. However, our analysis on the individual foods reported to be major contributors of phenolic acids showed no significant results, reinforcing the hypothesis that the potential benefits reported in literature may not depend on individual foods but on common compounds, such as ferulic and caffeic acids.

Nevertheless, these results should be taken with caution. In fact, caffeic and ferulic acid intake may simply be surrogates for the intake of fruits, vegetables and dietary fiber, which may be independently protective in the case of prostate cancer or protective as displacement component of the diet for more prostate carcinogenic foods (i.e., meats and dairy).

The findings of the present study should be considered in light of some limitations. First, the complexity of phenolic acids composition is complex. In fact, food frequency questionnaires which assess dietary habits may lead to measurement errors. It is not only due to recall bias but also to the estimation by using different food composition databases, which may not be complete for the whole range of foods consumed. Second, a common limitation of population-based case-control studies including non-screened population as controls is that we are unaware of whether some undiagnosed PCa occurred in the control group. However, the rate of potentially undiagnosed cancer would be low and not likely to affect the results of the study.

Further studies, reproducing the same experimental design and planning a specific personal dietary intake could be useful in better understanding the role of caffeic and ferulic acid in preventing PCa.

## 4. Materials and Methods

### 4.1. Study Population

As previously reported, we conducted a population-based case–control study on the association between prostate cancer and dietary factors was conducted from January 2015 to December 2016 in a single institution of the municipality of Catania, southern Italy [27]. Patients with elevated PSA and/or suspicious PCa underwent transperineal prostate biopsy (≥12 cores). A total of 118 histopathologically-verified PCa cases were collected.

Controls were selected from a sample of 2044 individuals included in a cohort study [28]: individuals were randomly selected among the same reference population of the cases, and matched by age, BMI, and smoking status with cases. A total of 222 controls were selected. All the study procedures were carried out in accordance with the Declaration of Helsinki (1989) of the World Medical Association and participants provided written informed consent after accepting to participate. The study protocol was approved by the ethic committee of the referent health authority (Registration number: 41/2015).

### 4.2. Data Collection

Demographics (including age, and educational level) and lifestyle characteristics (including physical activity, smoking and drinking habits) were collected. Educational level was categorized as: (i) low (primary/secondary); (ii) medium (high school); and (iii) high (university). Physical activity level was evaluated through the International Physical Activity Questionnaires (IPAQ) [29], which comprised a set of questionnaires (5 domains) investigating the time spent being physically active in the last seven days. Bsed on the IPAQ guidelines, final scores allowed categorizing physical activity level as: (i) low; (ii) moderate; and (iii) high. Smoking status was categorized as: (i) non-smoker; (ii) ex-smoker; and (iii) current smoker. Alcohol consumption was categorized as: (i) none; (ii) moderate drinker (0.1–12 g/day); and (iii) regular drinker (>12 g/day).

### 4.3. Dietary Assessment

**Figure 1:**
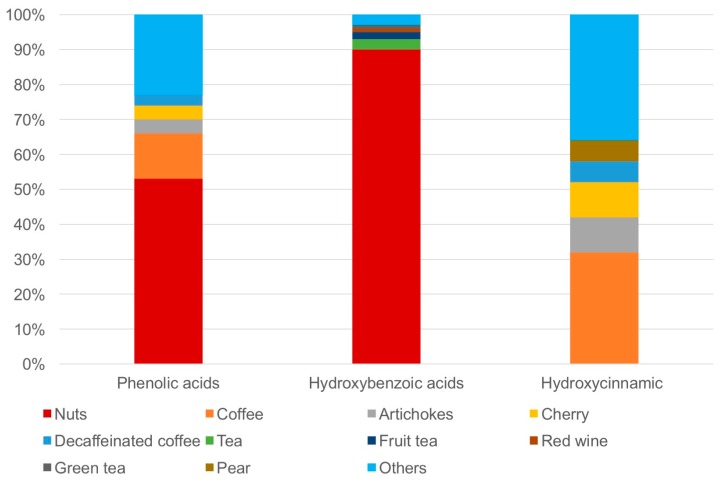
Distribution of major dietary sources of phenolics in the all cohort.

Dietary data were collected using two food frequency questionnaires (FFQs) specifically developed and validated for the Sicilian population [30,31]. Figure 1 shows the Distribution of major dietary sources of phenolics in the all cohort.

The long-version FFQ consisted of 110 food and drink items. Patients were specifically asked whether they changed their diet due to course of the disease and to answer to the questionnaire referring to their habitual diet before the disease. Participants were asked how often, on average, they had consumed foods and drinks included in the FFQ, with nine responses ranging from “never” to “4–5 times per day”. Intake of food items characterized by seasonality referred to consumption during the period in which the food was available and then adjusted by its proportional intake in one year.

### 4.4. Estimation of Polyphenol Intake

The methodology used to retrieve dietary polyphenols has been widely used in literature and largely described elsewhere [32]. Briefly, data on the polyphenol content in foods were obtained from the Phenol-Explorer database (www.phenol-explorer.eu). A new module of the Phenol-Explorer database containing information on the effects of cooking and food processing on polyphenol contents was used whenever possible to apply polyphenol-specific retention factors [33]. A total of 75 items were searched in the database after exclusion of foods that contained no polyphenols. Following the standard portion sizes used in the study, food items were converted in g or mL and then proportioned to 24-h intake. Then, a search was carried out in the Phenol-Explorer database to retrieve mean content values for phenolic acids (total, major subclasses and selected compounds) contained in the foods obtained and their intake was then calculated by multiplying the phenolic acid content by the daily consumption of each food. Finally, intake of phenolic acids, their subclasses and individual compounds was adjusted for total energy intake (kcal/day) using the residual method.

### 4.5. Statistical Analysis

Categorical variables are presented as frequencies and percentages, continuous variables are presented as means and standard deviations. Differences of frequency between groups were calculated by Chi-square test. Phenolic acid intake distribution was tested for normality distribution with the Kolmogorov–Smirnov test and it followed a slightly asymmetric normal distribution due to extreme values of the upper side. Mann–Whitney *U*-test and Kruskall–Wallis test were used to compare differences in intakes between groups, as appropriate. Association between dietary intake of phenolic acids and PCa was calculated through logistic regression analysis adjusted for age (years, continuous), energy intake (kcal/day, continuous), weight status (normal, overweight, obese), smoking status (smokers, non-smokers), alcohol consumption (<12 g/day, ≥12 g/day), physical activity level (low, medium, high), and family history of PCa. All reported *p* values were based on two-sided tests and compared to a significance level of 5%. SPSS 17.0 (SPSS Inc., Chicago, IL, USA) software was used for all the statistical calculations.

## 5. Conclusions

High intake of caffeic acid and ferulic acid may be associated with reduced risk of PCa. Diets rich in ferulic acid and caffeic acid may have beneficial effects in reducing PCa incidence. However, we are far away from suggesting dietary change based on our observation in the general population, and further clinical studies designed to modify serum concentrations of these compounds in subjects at risk of PCa should be undergone.

## Figures and Tables

**Table 1:** Baseline characteristics of cases and controls.

	Cases (*n* = 118)	Controls (*n* = 222)	*p*-Value
Age (y), mean (SD)	69.13 (6.60)	68.09 (8.18)	0.19
BMI, mean (SD)	26.49 (3.34)	27.49 (3.28)	0.30
Weight status, *n* (%)			
Normal	42 (35.6%)	59 (26.6%)	
Overweight	60 (50.8%)	127 (57.2%)	
Obese	16 (13.6%)	36 (16.2%)	
Smoking status, *n* (%)			0.15
Non-smoker	68 (57.6%)	143 (64.4%)	
Current smoker	50 (42.4%)	79 (35.6%)	
Alcohol intake, *n* (%)			0.16
<12 g/day	55 (46.6%)	153 (68.9%)	
≥12 g/day	63 (53.4%)	69 (31.1%)	
Education, *n* (%)			0.11
Primary/secondary	96 (81.4%)	49 (22.1%)	
High school/university	22 (18.6%)	173 (77.9%)	
Physical activity level, *n* (%)			0.21
Low	38 (32.2%)	49 (26.2%)	
Medium	64 (54.2%)	67 (35.8%)	
High	16 (13.6%)	71 (38.0%)	
Family history of prostatic cancer, *n* (%)	43 (36.44%)	9 (4.05%)	<0.01

SD = standard deviation; BMI = body mass index.

**Table 2:** Differences of mean total, subclasses and individual phenolic acids between cases and controls.

	Cases (*n* = 118), Mean SD	Controls (*n* = 222), Mean SD	*p*-Value
Total phenolic acids	383.41 (522.77)	400.20 (540.07)	0.78
Subclasses			
Hydroxybenzoic acids	218.46 (487.25)	238.29 (525.82)	0.73
Hydroxycinammic acid	164.04 (106.69)	160.95 (97.57)	0.78
Hydroxyphenilacetic acid	0.64 (0.56)	0.65 (1.07)	0.34
Individual phenolic acids			
Caffeic acid	2.76 (1.84)	2.28 (2.40)	0.05
Cinnamic acid	0.48 (0.60)	0.57 (1.81)	0.60
Vanillic acid	0.52 (0.38)	0.46 (0.59)	0.34
Ferulic acid	4.04 (3.35)	2.80 (2.57)	<0.001

SD = standard deviation.

**Table 3:** Association between quartiles of total, subclasses and individual phenolic acid intake and prostate cancer.

	Phenolic Acid Quartiles, OR (95% CI)			
	Q1	Q2	Q3	Q4
Total phenolic acids				
No. of cases	26	35	26	31
OR (95% CI) ^a^	Ref.	1.30 (0.68–2.47)	0.87 (0.44–1.69)	0.81 (0.41–1.62)
OR (95% CI) ^b^	Ref.	1.21 (0.48–3.00)	0.65 (0.24–1.69)	1.02 (0.37–2.78)
Hydroxybenzoic acids				
No. of cases	31	21	32	34
OR (95% CI) ^a^	Ref.	0.70 (0.35–1.37)	0.99 (0.53–1.86)	0.88 (0.47–1.67)
OR (95% CI) ^b^	Ref.	0.77 (0.30–2.01)	0.46 (0.18–1.18)	0.75 (0.29–1.94)
Hydroxycinammic acid				
No. of cases	28	28	38	24
OR (95% CI) ^a^	Ref.	0.86 (0.44–1.67)	1.02 (0.54–1.92)	0.56 (0.27–1.13)
OR (95% CI) ^b^	Ref.	1.60 (0.64–3.99)	1.54 (0.60–3.94)	0.76 (0.27–2.13)
Hydroxyphenilacetic acid				
No. of cases	17	16	47	38
OR (95% CI) ^a^	Ref.	0.83 (0.38–1.82)	2.62 (1.33–5.17)	1.61 (0.79–3.28)
OR (95% CI) ^b^	Ref.	0.61 (0.24–1.58)	1.66 (0.71–3.88)	0.77 (0.29–2.07)
Caffeic acid				
No. of cases	8	14	44	52
OR (95% CI) ^a^	Ref.	1.42 (0.71–2.83)	0.74 (0.39–1.43)	0.28 (0.13–0.58)
OR (95% CI) ^b^	Ref.	1.86 (0.65–5.29)	0.79 (0.30–2.08)	0.32 (0.11–0.87)
Cinnamic acid				
No. of cases	30	29	25	34
OR (95% CI) ^a^	Ref.	0.87 (0.45–1.65)	0.76 (0.39–1.48)	0.81 (0.42–1.54)
OR (95% CI) ^b^	Ref.	1.29 (0.52–3.20)	0.69 (0.28–1.73)	1.06 (0.39–2.83)
Vanillic acid				
No. of cases	5	24	53	36
OR (95% CI) ^a^	Ref.	1.84 (0.92–3.67)	0.99 (0.50–1.94)	0.53 (0.25–1.11)
OR (95% CI) ^b^	Ref.	0.95 (0.33–2.73)	0.54 (0.20–1.46)	0.30 (0.10–0.85)
Ferulic acid				
No. of cases	19	12	36	51
OR (95% CI) ^a^	Ref.	1.51 (0.77–2.98)	0.65 (0.33–1.25)	0.50 (0.26–0.97)
OR (95% CI) ^b^	Ref.	1.62 (0.60–4.37)	0.63 (0.25–1.60)	0.44 (0.17–1.10)

OR, odds ratio; CI, confidence interval; Ref, Reference; ^a^ ORs were adjusted for energy intake (kcal/dayay, continuous); ^b^ ORs were adjusted for age (years, continuous), energy intake (kcal/day, continuous), weight status (normal, overweight, obese), smoking status (smokers, non-smokers), alcohol consumption (<12 g/day, ≥12 g/day), physical activity level (low, medium, high), and family history of prostatic cancer.

**Table 4:** Association between quartiles of total, subclasses and individual phenolic acid intake and advanced prostate cancer.

	Phenolic Acid Quartiles, OR (95% CI) ^a^			
	Q1	Q2	Q3	Q4
Total phenolic acids	Ref.	0.75 (0.16–3.55)	0.68 (0.14–3.21)	0.34 (0.05–2.22)
Subclasses				
Hydroxybenzoic acids	Ref.	0.52 (0.09–2.97)	0.43 (0.50–0.09)	0.85 (0.86–0.20)
Hydroxycinammic acid	Ref.	0.44 (0.10–1.86)	0.25 (0.05–1.32)	0.10 (0.01–1.10)
Hydroxyphenilacetic acid	Ref.	0.45 (0.04–5.18)	3.40 (0.67–17.22)	0.71 (0.09–5.66)
Individual phenolic acids				
Caffeic acid	Ref.	0.62 (0.14–2.74)	0.31 (0.07–1.33)	0.01 (0.00–1.00)
Cinnamic acid	Ref.	0.18 (0.02–1.61)	0.57 (0.13–2.53)	0.46 (0.10–2.15)
Vanillic acid	Ref.	1.58 (0.35–7.11)	0.69 (0.14–3.43)	0.13 (0.01–1.55)
Ferulic acid	Ref.	1.51 (0.32–1.10)	1.66 (0.97–2.83)	1.66 (0.97–2.83)

OR, odds ratio; CI, confidence interval; Ref, Reference; ^a^ ORs were adjusted for energy intake (kcal/day, continuous).
